# Primitive uterine neuroectodermal tumours: Two case reports

**DOI:** 10.4274/tjod.58295

**Published:** 2015-09-15

**Authors:** İpek Eskiyörük, Ümran Küçükgöz Güleç, Semra Paydaş, Ahmet Barış Güzel, Mehmet Ali Vardar, Emine Bağır

**Affiliations:** 1 Çukurova University Faculty of Medicine, Department of Obstetrics and Gyneacology, Adana, Turkey; 2 Çukurova University Faculty of Medicine, Department of Medical Oncology, Adana, Turkey; 3 Çukurova University Faculty of Medicine, Department of Pathology, Adana, Turkey

**Keywords:** Immunohistochemistry, primitive neuroectodermal tumors, uterus

## Abstract

Primitive neuroectodermal tumor (PNET) arise from Kulchitsky cells and are rarely seen in the female genital tract. Differential diagnosis of PNET can be made based on immunohistochemical profiles and genetic analyses. Genital tract pNETs are very aggressive pathologies with different clinical and molecular manifestations and there are no standard guidelines for treatment. We aimed to present two cases of uterine PNETs with different symptomatology and clinical findings.

## INTRODUCTION

Primitive neuroectodermal tumors (PNET) of the female genital tract are rarely observed. The term PNET was first used by Hart and Earle in 1973 to introduce a group of tumors derived from fetal neuroectodermal cells^(1)^. These tumors were noted to have morphologic features of small round cell tumors with variable degrees of neural, glial, and ependymal differentiation. Ewing’s sarcoma and primitive neuroectodermal tumor (PNET) stand for a single group of bone and soft tissue tumours in which PNET with evidence of neural differentiation lies at one end of the spectrum, and undifferentiated Ewing’s sarcoma lies at the other. Both have similar phenotypes and share an identical chromosomal translocation. There are two main classifications of PNET, including central and peripheral, depending on the origin and localization of the cell. Central PNETs are derived from the neural tube and primarily involve the brain and spinal cord. The peripheral PNETs originate from the neural crest and occur outside the central nervous system, often involving the sympathetic nervous system or soft tissues and bones^(2)^. Pelvic PNETs are frequently observed in the uterine corpus, ovaries, cervix, and vulva. PNET can be diagnosed based on histologic examinations. Also, immunohistochemical profiles and genetic analyses commonly help the pathologist to differentiate between PNET and other small round cell tumors. Nowadays, the cornerstone treatment of PNET is surgery followed by chemotherapy and/or radiotherapy^(2)^. This study aimed to describe and discuss this rare pathology of the female genital tract.

## CASE REPORT

**Figure 1:**
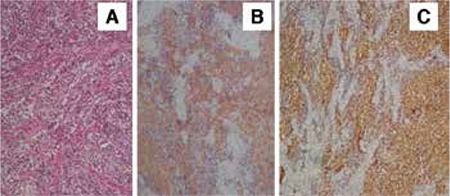
Case 1. A) Small round tumor cells were observed with scanty cytoplasm with myometrial invasion and without rosette formation (HEx200), B) Immunohistochemical findings of tumor cells. The neoplastic cells are diffuse positive for CD56, C) The neoplastic cells are diffuse positive for NSE

A woman aged 63 years, gravida 7, para 3, presented to our clinic with a two-month history of vaginal bleeding. She had been postmenopausal for 10 years. She had a normal physical examination findings with a mass-like submucous degenerated myoma nodule in the uterine cavity on ultrasonographic examination. An endometrial biopsy and papanicolaou (PAP) smear had been performed at another center. The endometrial biopsy was reported as endometrial hyperplasia with cellular atypia and the PAP smear was normal. The patient’s pathology report was from another center and there was no data about its type. We did not perform frozen section. Laparascopic hysterectomy and bilateral salpingo-oopherectomy was performed. A 4x2.5 cm polypoid mass pressing the myometrium, localized at the site of low and left lateral uterine segment was observed in the cavity during macroscopic evaluation. No special abnormality was observed in the cross sectional view of the ovaries. The tumor had entirely penetrated the wall of the uterus and lymphovascular and cervical invasion were observed histologically. The tumor’s growth pattern was diffuse and it had relatively uniform neoplastic cells, without rosette formation. A large area of necrosis was seen. There was no associated endometrial pathology in the hysterectomy specimen sections. The tumor cells showed scant cytoplasm and hyperchromatic nuclei. Mitoses and numerous apoptotic bodies were found. Immunonegative results were determined for LCA, TTF-1, Cytokeratin, Desmin, CD99, CD10, Myogenin, NFP, FLI1, and chromogranin for differential diagnosis of other small round cell tumors. The tumor cells showed positive immunoreactivity for synaptophysin, NSE, and CD56 (Figure 1). The final diagnosis was PNET after microscopic and immunohistochemical studies. Malignancy level hypermetabolic lymph nodes were observed in PET images of the left iliac chain in the postoperative period. After the patient was considered as Federation of Gynecology and Obstetrics (FIGO) Stage IIIC, we decided to administer the EVAIA chemotherapy protocol (ifosfamide, dactinomycin, adriamycin, etoposide, vincristine, uromitexan, and G-CSF). However, severe renal failure and granulocytopenic fever developed after 3 cycles. For this reason chemotherapy was changed to a VAC-IE combination and the patient is currently in remission.

**Figure 2:**
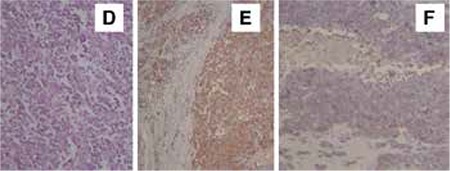
Case 2. D) Small round tumor cells were observed with scanty cytoplasm composing rosette formation and with myometrial invasion (HEx200), E) Immunohistochemical findings of tumor cells. The neoplastic cells are diffuse positive for NSE, F) The neoplastic cells are positive for CD99

The second case was found in a girl aged 17 years who presented to an outside clinic with a three-month history of vaginal bleeding. She was underwent surgery for myoma uteri in that clinic. The extracted material included four pieces of myoma, the largest was 6x5x3.5 cm, and the smallest was 1.3x0.6x0.1 cm. The largest myoma nodule had an irregular surface, elastic form, and was brown grey-white in color. The histopathologic diagnosis of PNET was made by our pathology department after small round tumor cells with rosette formation were observed with myometrial invasion containing wide areas of necrosis. In the immunohistochemical studies, specimens had a characteristic positive immunoreactivity for CD99, Vimentin, and NSE, and negative for LCA, Desmin, CD34, Cytokeratin, CD10 (Figure 2). There was no abnormal findings in scanning abdominopelvic magnetic resonance imaging (MRI) and thorax compterized tomography (CT). We scheduled surgery but the patient did not attend and consequently left follow-up. When we later had a review with the patient, we learned that she had undergone surgery at another center (hysterectomy and salpingo-oopherectomy) and received chemotherapy (VAC and ifosfamide-etoposide for 52 weeks). The treatment ended in 2010 and she has been in remission since November 2014.

## DISCUSSION

The most frequent localization of PNETs in female genital tract is reported to be the ovaries^(2)^. The cervix and vulva are rarely the primary site^(3)^. PNET of uterus has been rarely reported. Neuroectodermal differentiation is a very unusual finding in uterine tumours; these tumours involve the endometrium and occasionally the myometrium. The age range of these patients is 12-81 years with a mean of 51 years^(2)^. There appears to be a bimodal age distribution with tumors either presenting in adolescence or in the postmenopausal period. Presenting symptoms included abnormal vaginal bleeding, uterine enlargement due to presumed leiomyomas, and pelvic mass. Patients mostly present with abnormal vaginal bleeding or postmenapousal bleeding, like in our cases. However, atypic presentations such as uterine rupture have also been reported^(4)^. Some of the uterine PNETs are associated with other tumor components such as endometrioid adenocarcinoma, carcinosarcoma, unclassified sarcoma, rhabdomyosarcoma, and adenosarcoma^(3)^. Our cases were not associated with other tumor components but 15 cases with other associated uterine pathologies were reported in the last review article^(2)^. There are different ideas regarding the origin of this tumor in the uterus^(4)^. These include prior implantation of fetal tissue, neo-metaplasia, malignant mixed Müllerian tumor with one-sided differentiation. Our first patient had had seven pregnancies but the second patient was nulliparous, thus there is no consensus on this subject, which remains controversial.

The differential diagnosis of uterine PNETs include tumors that exhibit neuroectodermal elements found in the central nervous system, including mature glial tissue of the endocervix or endometrium, immature teratoma with glial tissue, pure uterine gliomas, carcinosarcoma with neuroectodermal differentiation, and retinal anlage tumor. The diagnosis is based on light microscopic and immunohistochemical evidence of neuroectodermal differentiation, with markers. The diagnosis of PNETs is difficult using routine hematoxylin and eosin staining because of features that overlap with other small cell tumors (non-Hodgkin’s lymphoma, small cell carcinoma, rhabdomyosarcoma, endometrial stromal sarcoma). Some immunohistochemical markers can be used for differential diagnosis of these tumors^(3)^. CD99 is useful but it is not a specific marker for PNET. For the first case, we found that CD99 was negative; however, it was positive in the second case. Furthermore, both cases had positive immunohistochemical reactivity for NSE as a neuroendocrine marker. Muscular (desmin-myogenin) and lymphoid markers (LCA) were negative in both cases.

Genetic tests in molecular pathology can identify translocations that help distinguish peripheral PNETs from other round cell tumors. Although peripheral PNETs generally have t(11;22) (q24;q12) chromosomal translocation, which leads to a chimeric transcript EWSR1/FLI1, it is not usually demonstrated in central type PNETs^(5)^. EWSR 1 means Ewing Sarcoma breakpoint region, also known as EWS. We did not perform any genetic studies, but of the 20 reported cases that have been evaluated for EWSR1 rearrangement, only 7 had a positive result. ESWR1 was more frequently found in younger patients (<50 years) according to this report^(2)^. Euscher et al.^(3)^ concluded that only tumors with PNET histology but lacking the ESWR1 translocation should be designated as central-type PNET.

For prognosis, FIGO’s annual report states that the two-year survival for uterine PNET is 46%^(6)^. Bartosch et al.^(2)^ demonstrated that almost all patients who have higher stage disease die in two years. Appropriate surgery and chemotherapy prolong the survival of patients. Surgical treatment consisting of hysterectomy and bilateral oophorectomy with or without lymphadenectomy should be performed, depending on the findings at surgery. No significant difference in survival has been found related with ESWR1 reearrangement. The prognosis of uterine PNETs seems to related with surgical stage, like with uterine carcinomas, but the survival rate is much worse. The 2-year survival rate was found as 68% for stage 1, 33% for stage 2, 58% for stage 3 and 0% for stage 4 in a review article^(2)^. Adjuvant chemotherapy and/or radiotherapy usually follows surgery^(7)^. The chemotherapy regimen may be chosen based on trials of bone PNETs but there is no consensus on an optimal chemotherapy regimen^(8)^. The most commonly used combinations are EVAIA and VAC; there has been no comparative trials for these regimens.

In view of our reported cases and the literature, there are not yet any standard guidelines for the treatment of genital tract PNETs.
